# Health Transformation Project and Defensive Medicine Practice among Neurosurgeons in Turkey

**DOI:** 10.1371/journal.pone.0111446

**Published:** 2014-10-21

**Authors:** Ihsan Solaroglu, Yusuf Izci, H. Gokce Yeter, M. Mert Metin, G. Evren Keles

**Affiliations:** 1 Koç University, School of Medicine, Department of Neurosurgery, Istanbul, Turkey; 2 Gulhane Military Medical Academy, School of Medicine, Department of Neurosurgery, Ankara, Turkey; 3 Koç University, School of Medicine, Istanbul, Turkey; Fraunhofer Institute for Cell Therapy and Immunology, Germany

## Abstract

**Background:**

The term “Defensive” medicine was coined in the early 1970′s and has been an important topic of scientific investigation and professional debate ever since.

**Objective:**

The aim of this study was to investigate the characteristics of defensive medicine, its reasons, and the extent to which it is practiced in the Turkish health care system. This is the first national survey to study the practice of defensive medicine among neurosurgeons in Turkey.

**Methods:**

The present cross-sectional study on defensive medicine assessed neurosurgeons registered at the Turkish Neurosurgical Society, who are actively working in various centers and hospitals within the Turkish health care system. A 40-question survey was adapted from existing measures described in the literature and was completed by a total of 404 neurosurgeons, representing 36.7% of the neurosurgeons registered at the Turkish Neurosurgical Society.

**Results:**

Seventy-two percent of the participants in the current study reported practicing defensive medicine. This practice was mainly reported among inexperienced neurosurgeons (74.4%). Most were younger than 40 years of age (75.2%), working in state hospitals/universities (72.7%), and living in the Marmara region (38%). Respondents reported engaging in defensive medicine by avoiding high-risk surgery (62.6%), ordering additional imaging studies (60.9%) and laboratory tests (33.7%), and referring patients to consultants (31.2%). Most participants consider every patient as a potential threat in terms of a medical lawsuit (68.3%) and do not believe the courts can distinguish malpractice from complications (89.6%).

**Conclusion:**

Concerns and perceptions about medical liability lead neurosurgeons to practice defensive medicine. By avoiding high-risk surgery, ordering unnecessary diagnostic tests, and referring the patients to consultants, neurosurgeons try to minimize the risk of malpractice and protect themselves from legal risks, resulting in higher healthcare expenditure and longer treatment periods.

## Introduction

Defensive medicine is defined as medical practices that help doctors avoid liability without providing any additional benefit to the patient [1]. More specifically, doctors practice defensive medicine owing to concerns about liability risk, and this contributes to an increase in healthcare expenditure [2], [3]. Several studies have focused on the effects of defensive medicine on doctors and patients. This is a major factor that increases the cost of medical care [4]–[7]. The cost of defensive medicine in the US has been estimated at $200 billion per year by the accounting firm Price Waterhouse [8] and as much as $5 billion per year by the Congressional Budget Office [9].

Malpractice liability affects all medical practitioners, but specific specialties such as emergency medicine, orthopedic surgery, neurosurgery, obstetrics/gynecology, and radiology, are particularly “high-risk” for litigation [6]. Neurosurgery is considered a “high-risk” specialty in medicine because of the necessity for acute decision-making in a significantly high proportion of cases, the small margin for error, and the potential for adverse outcomes. As malpractice liability continues to be a concern, neurosurgeons are offering fewer treatment options to mitigate liability exposure [10], [11].

The local frequency and economic impact of defensive medicine among neurosurgeons are not known in Turkey, which is one of the leading countries in the field of neurosurgery in Europe. The current study aimed to investigate the extent and characteristics of the practice of defensive medicine in this high-risk specialty.

## Methods

IRB Approval from Koç University Committee on Human Research was obtained before starting the survey. The purpose of this study was disclosed to the participants prior to beginning the survey.

A 40-question survey comprising previously validated questions was compiled from earlier studies [6], [11]. This survey included questions on five basic domains that have been found to influence defensive practices: (1) surgeons' demographic features; (2) patient healthcare system; (3) malpractice premiums of the surgeons; (4) perceptions of surgeons related to liability; (5) defensive medicine practice. The survey took about 10 min on average to complete. In a preliminary assessment, focused groups were established and the survey was administered to a small group of 20 neurosurgical practitioners. Based on feedback, the survey was finalized and an online questionnaire was established. The online survey was sent to 1100 members of the Turkish Neurosurgical Society (TNS) who have a valid email address. The TNS is the largest neurosurgical society in Turkey and represents more than 95% of neurosurgeons in military, academic, and private practices. The survey respondents consisted of neurosurgeons in different practice settings including state hospitals, military hospitals, university hospitals, and private practice. The link to the survey was presented to TNS members via e-mail. After providing consent, participants completed the survey online. The study was conducted over a 2-month period.

The participants were divided into two groups according to the duration of neurosurgery practice (experience), age, gender, and the number of operation performed per year. The term “experienced” neurosurgeons was operationally defined as neurosurgery practice for more than 11 years after residency. The participants were also classified according to their location (geographic region in Turkey), institution, subspecialty, and malpractice premium. The perceptions of defensive medicine practiced by these groups were compared using Pearson's Chi-square tests.

## Results

### Respondents' profile

**Figure 1:**
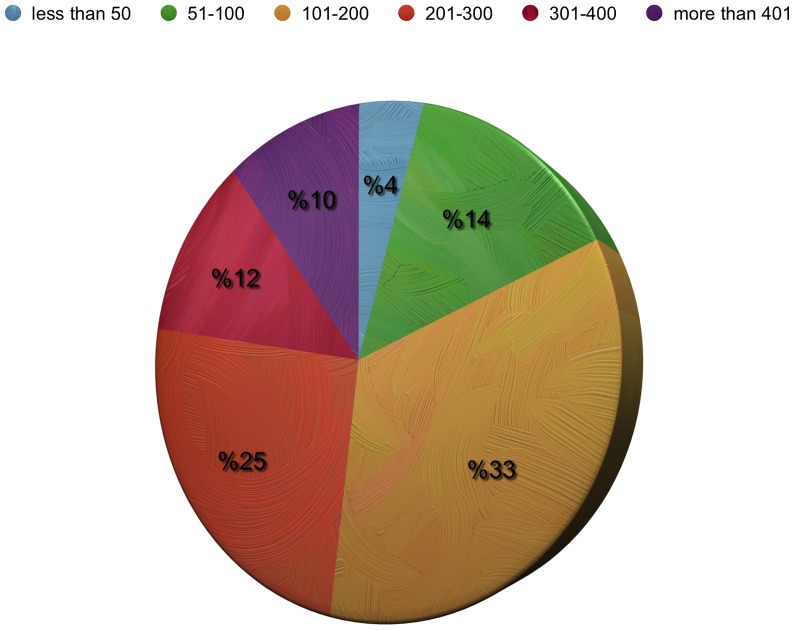
Distribution of the respondents according to number of operation per year.

**Figure 2:**
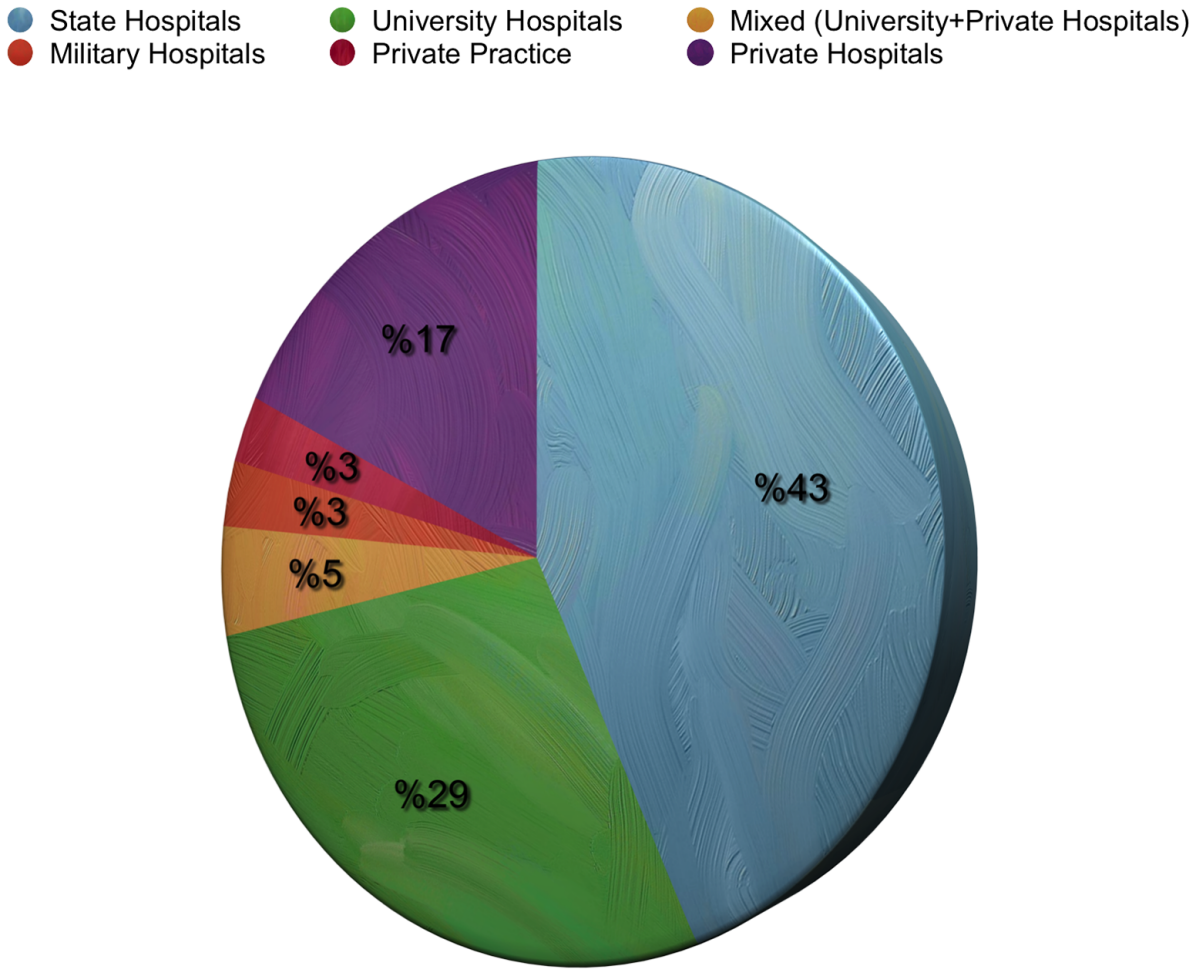
Distribution of the respondents according to the hospital where they work.

**Figure 3:**
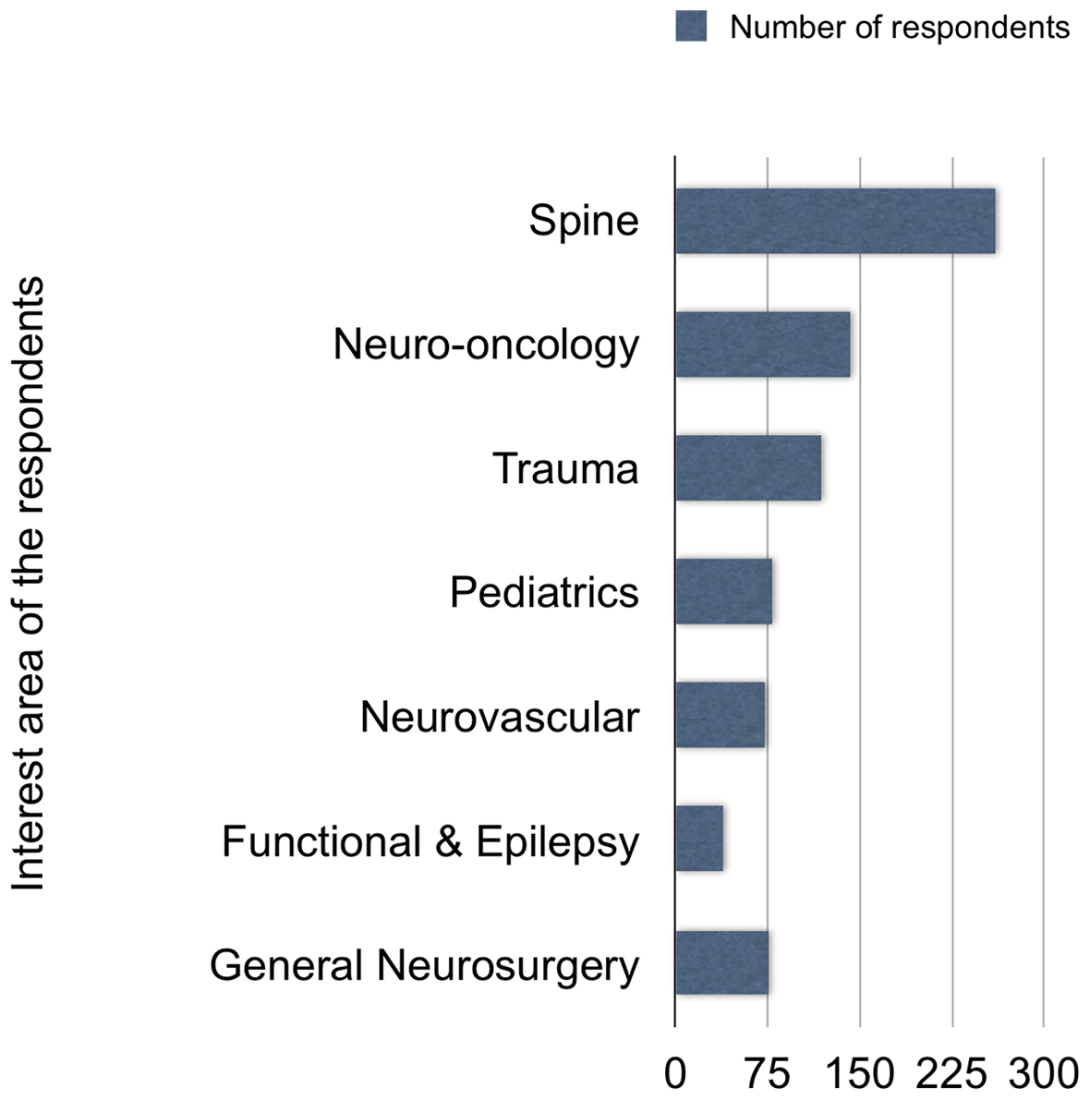
Distribution of the respondents according to their area of interest.

Of the 1100 neurosurgeons currently working in Turkey and registered by the TNS, 404 (36.7%) completed and returned the questionnaire. Ninety-five percent (*n* = 384) were men and five percent (*n* = 20) were women. One hundred and eighty-two (45%) were 40–49 years of age and 152 (37.6%) neurosurgeons had a post-specialization experience of 11–20 years. One hundred and twenty-eight (31.7%) respondents reported working in the Marmara region and 107 (26.5%) in the Central Anatolia region. The annual number of operations was 101–200 for 135 (33.4%) of the participants, and 201–300 for 103 (25.5%) of the participants (**Figure 1**). One hundred seventy-four (43%) were working at state hospitals, and 116 (28.7%) at university hospitals (**Figure 2**). The interest areas were reported as: spinal and peripheral nerve surgery in 260 (64.4%) of the participants, neuro-oncology in 142 (35.1%), trauma in 118 (29.2%), and pediatric neurosurgery in 78 (19.3%) (**Figure 3**). The survey covered 36.7% of Turkish neurosurgeons, whose characteristics are shown in **Table 1** **.**

**Table 1:** Characteristics of neurosurgeons based on sex, age groups, and experience.

	Number	Percentage (%)
**Sex**		
Male	384	95
Female	20	5
**Age groups**		
30–9	126	31.2
40–49	182	45.0
50–59	73	18.1
60–69	19	4.7
70 and older	1	0.2
**Experience in neurosurgery**		
Less than 5 years	35	8.7
5–10 years	100	24.8
11–20 years	152	37.6
21–30 years	86	21.3
More than 30 years	28	6.9
**Regions**		
Mediterranean Region	44	10.9
Eastern Anatolia Region	21	5.2
Aegean Region	49	12.4
Southeast Anatolia Region	29	4.7
Central Anatolia Region	107	26.5
Black Sea Region	34	8.4
Marmara Region	128	31.7

### Healthcare insurance of the respondents' patients

Social security administration was the main healthcare provider for more than 75% of the patients of the respondents, and less than 10% of the patients had private insurance.

### Malpractice premium

**Table 2** displays information related to malpractice premiums in the context of changes to payments and malpractice premiums as a percentage of the physician income. The malpractice premium was paid by the hospital and the physician in the case of 327 (80.9%) respondents, by the hospital for 11, and by the physician in the case of 58 respondents. The proportion of malpractice premium to annual income was less than 10% for 299 (74%) respondents. The malpractice premium had not changed during the last 3 years for 104 (25.7%) respondents.

**Table 2:** Distribution of the participants based on the malpractice premiums.

	Number	Percentage (%)
**Compensation of malpractice insurance premium**		
By the hospital	11	2.7
By the hospital and physician	327	80.9
By the physician	58	14.4
**Percent of the malpractice premium to the annual income**		
More than 60%	5	1.2
50–59%	7	1.7
40–49%	5	1.2
30–39%	4	1.0
20–29%	11	2.7
10–19%	47	11.6
Less than 10%	299	74.0
**Changes in the malpractice premiums during the last 3 years**		
Increased more than 25%	28	6.9
Increased 10–25%	65	16.1
Increased less than 10%	48	11.9
Not changed	108	26.7
Decreased less than 10%	13	3.2
Decreased 10–25%	22	5.4
Decreased more than 25%	4	1.0
Unknown	95	23.5

### Perceptions of the neurosurgeons

Most of the neurosurgeons (*n* = 333, 82.4%) believed that a severe crisis relating to medical liability exists in their specialty. According to the majority of respondents, medical liability affects the preferences of the neurosurgeons depending on the geographic region in which they live (*n* = 251) and the duration of practice (*n* = 304). Two hundred seventy-six (68.3%) of the respondents considered their patients in terms of a potential lawsuit. Moreover, the ways of preventing a malpractice lawsuit were perceived to include the following: informing patients about all the risks of surgery (*n* = 277, 68.6%), improving the quality of healthcare (*n* = 207; 51.2%), changing the current codes on malpractice (*n* = 166, 41.1%), and decreasing the expectations of the patients (*n* = 101, 25%) (**Table 3**).

**Table 3:** Perceptions of the neurosurgeons who participated this survey.

	Number	Percentage (%)
**“There is a medical liability crisis in my area”**		
Strongly Agree	214	53.0
Agree	119	29.5
Neutral	32	7.9
Disagree	17	4.2
Strongly Disagree	7	1.7
**“I view every patient as a potential lawsuit”**		
Strongly Agree	137	33.9
Agree	139	34.4
Neutral	34	8.4
Disagree	68	16.8
Strongly Disagree	17	4.2
**“What are the ways to prevent potential lawsuits”**		
Inform the patients about the all risks of surgery	277	68.6
Improve quality of healthcare regardless of the cost	207	51.2
Reduce patients expectancies	101	25.0
Punish doctors who are responsible for malpractice	12	3.0
Change the current codes on malpractice	166	41.1
Become transparent on all medical procedures	161	39.9

### Defensive medicine practices

Two hundred ninety-two (72.3%) respondents believed that they practice defensive medicine. Neurosurgeons who believed that they perform defensive medical practices were less experienced (74.4%) and less than 40 years old (75.2%) (*p* = 0.02 for both groups). There was no statistically significant difference among the types of institutions and the geographic regions of the neurosurgeons (*p* = 0.083, and *p* = 0.732 respectively), and no statistically significant difference in the frequency of reports by male and female neurosurgeons (*p* = 0.252) with respect to their practice of defensive medicine. The practices done solely to minimize the risk of a lawsuit were as follows: avoiding high-risk procedures (*n* = 253, 62.6%), ordering additional imaging studies (*n* = 246, 60.9%) and laboratory tests (*n* = 136, 33.7%), and referring to other hospitals (*n* = 126, 31.2%). Malpractice liability premiums were considered a “minimal” burden by 55.2% of respondents. Most participants did not believe that the courts could distinguish malpractice from complications (89.6%) (**Table 4**).

**Table 4:** Defensive medicine practices among the respondents.

	Number	Percentage (%)
**“I believed that I perform defensive medicine practice”**		
Strongly Agree	104	25.7
Agree	188	46.5
Neutral	35	8.7
Disagree	58	14.4
Strongly Disagree	9	2.2
**Defensive medicine practices**		
Order lab tests	136	33.7
Refer the patients to other medical centers	126	31.2
Prescribe medications	20	5.0
Suggest an invasive procedure to confirm diagnosis (e.g., biopsies)	31	7.7
Order radiological exams	246	60.9
Avoid certain procedures or interventions	176	43.6
Stop practicing or eliminate high-risk procedures	253	62.6
**Overall burden of malpractice insurance**		
Not a burden	67	16.6
Minor burden	223	55.2
Moderate burden	64	15.8
Major burden	28	6.9
Extreme burden	7	1.7
No idea	3	0.7

## Discussion

Defensive medicine is common, especially in the United States of America. Rates are as high as 79% to 93%, particularly in emergency medicine, obstetrics, and other high-risk specialties [2], [6]. It has been reported that fear of litigation and loss of reputation are the major causes of defensive medicine [12].

There are two types of defensive medicine. Positive defensive medicine is expressed by the increased use of resources, both to reduce the risk of receiving a further complaint and to increase doctors' ability to defend one; this could be called “augmented” or “extra” medical practice [13]. When neurosurgeons perform extra tests or procedures primarily to reduce their malpractice liability, they are practicing positive defensive medicine. Negative defensive medicine refers to a withdrawal of medical services; for example, neurosurgeons may avoid certain patients or surgical procedures if they believe these place them at greater risk for litigation [13], [14]. Nahed et al. performed the largest study on the practice of defensive medicine among 1028 neurosurgeons in the United States and reported that some neurosurgeons have eliminated high-risk procedures to minimize malpractice risk [11]. Our results are similar to those of neurosurgeons in the United States. Most neurosurgeons in Turkey practice negative defensive medicine by avoiding high-risk surgery. Although this does not increase the cost of healthcare, patients are negatively affected by the practice. This kind of practice at teaching and/or university hospitals may also negatively affect the resident training programs. University and training hospitals should be the primary centers for high-risk and complicated surgery, but current numbers show the opposite due to new regulations on healthcare in Turkey. The most significant long-term effect would be that residents have inadequate and inefficient training without the opportunity to observe or perform certain operations. Sub-specialty training programs would also be impaired due to this avoidance by trainers. Subsequently, the number of patients receiving proper treatment would be lower. Moreover, lack of motivation may also decrease the academic productivity of faculty members.

The Health Transformation Project was put into effect in 2003 by The Ministry of Health of the Republic of Turkey, which prohibited private practice by doctors working in all hospitals connected to the ministry including university hospitals, training hospitals, and state hospitals. In addition, the incomes of the doctors working in these hospitals were regulated in accordance with a “Performance Based Salary System,” in which every procedure is allocated a constant wage that assigns the quantity, but not the quality of work as the primary basis for determining salaries. Hence, protracted and complicated surgery became unfavorable for neurosurgeons. At the same time as the Health Transformation Project, Turkey introduced malpractice laws that effectively increased this tendency to avoid protracted and high-risk surgery, because of the absence of special malpractice courts.

Perhaps the greatest irony is that defensive medicine may be counterproductive and might actually increase the malpractice risk [15]. Studdert et al. has suggested that providing aggressive treatment for low-risk conditions or ordering tests and performing diagnostic procedures with low predictive values may become the legal standard of care [6]. Moreover, serious violations of the standard of care such as unnecessary invasive procedures and surgery could be the basis for malpractice litigation. Although doctors must act to avoid unnecessary utilization of tests and procedures, liability concerns greatly affect doctors' decision-making in ordering tests because of malpractice claims for misplaced or delayed diagnoses. Kachalia et al. have suggested that the malpractice law could create a vicious circle for doctors: “the more their colleagues practice defensive medicine, the more legally vulnerable they become if they do not” [16].

Unnecessary tests and invasive procedures not only increase the risks but also the cost of healthcare. High levels of monitoring and testing would seemingly be acceptable to healthcare providers, patients, authorities, and payers if those measures assured better results [2]. The risks from neurosurgery have been dramatically reduced by better preoperative imaging and surgical techniques, including improved imaging technology such as neuronavigation systems, intraoperative computed tomography, and magnetic resonance imaging, but there is no evidence that this increased reliance on more aggressive monitoring and intervention is applicable across all fields of medicine.

According to the Ministry of Finance Budget Justification for 2013 Report, public pharmaceutical and curative health services expenditure dramatically increased from 17.6 billion TL per year to 47.7 billion TL per year between 2005 and 2012 in Turkey [17]. Although there is no data to estimate the total cost of defensive medicine practice, ordering imaging studies and additional laboratory tests may increase healthcare expenditure, especially for neurosurgery practice. It is also ethically wrong to order any test that involves risk, cost, burden, or dignitary harm to a patient solely for personal benefit [16]. A question has always been raised: “How does one determine that a particular test is unnecessary?” This is one of the important limitations of surveys on defensive medicine practice.

Asking doctors directly in surveys, or linking differences in their actual procedure utilization rates to differences in their risk of liability, are ways to estimate whether and how frequently procedures are used for defensive reasons [2]. It is very difficult to measure defensive medical practice accurately because of the limitations of these measurement techniques. Doctors may be inclined to respond with the answer most likely to elicit a favorable political response that exaggerates their true level of concern about malpractice. They may also justify their choices purely on clinical grounds when other factors are in fact operating [2].

Aynacı performed a large study on defensive medicine in Turkey [18] among 762 doctors from different specialties. All participants of this survey were from the Central Anatolia region of Turkey. Aynaci showed that the doctors did practice defensive medicine. He found that the doctors at state hospitals, surgeons, practitioners at emergency services, doctors who were sued for malpractice, those who had malpractice insurance, those who had concerns after the new Turkish penal code had been enacted, and those who thought that they were at high risk of encountering a malpractice claim at any moment were more likely to practice positive and negative defensive medicine, and the difference was statistically significant [18]. Practicing defensive medicine was related to the concerns of the subjects, which put doctors at risk. Although this was a well-designed study, the data obtained was insufficient to develop or contribute to generalizable knowledge regarding defensive medicine practice among neurosurgeons in Turkey. Among 762 doctors included in the study, there were only 24 neurosurgeons, which represents 3.2% of research subjects. As in the current study, neurosurgeons expressed their concerns about practices after the new Turkish penal code was implemented and emphasized that the courts could not distinguish malpractice from a complication when a lawsuit occurs.

There are various strategies could improve the current system and prevent defensive medical practice among neurosurgeons. Firstly, malpractice cases should be removed from the regular court system and special malpractice courts should be created. In Turkey, most neurosurgeons do not believe that the courts can distinguish malpractice from complications (89.6%). Secondly, creating a forum to discuss malpractice cases will disseminate information to medical and law communities. This will help to determine what went wrong and identify where changes may be needed [19]. Finally, making the compensation process for medical injury less adversarial and distressing have also been suggested in the literature to reduce liability fears among neurosurgeons [16].

Our study has several limitations that should be taken into consideration when the findings are interpreted and extrapolated. Firstly, the major limitation is that the number of respondents is small. Although we tried to reach all the neurosurgeons in Turkey, only 404 (36.7%) of them completed and returned the questionnaire. Hence, the results may not be generalizable to all neurosurgeons in Turkey. However, when the diverse distribution of the respondents according to number of operation per year, hospitals in which they are working, and their area of interest is taken consideration, our national study has achieved the best possible insight into the practice of defensive medicine in Turkey. Secondly, it is quite difficult to find an objective method for measuring defensive medicine among neurosurgeons in Turkey. Our data are based on self-report, and the information may be inaccurate. The distinction between inappropriate and appropriate care is not clear in many clinical situations, especially in cases where acute intervention is required. Another problem is the difficulty in distinguishing between liability-related motivators and other factors that influence clinical decision-making, as suggested by Asher et al. [20]. On the other hand, self-reports of defensive medicine may be biased, and may lead doctors to overstate the frequency of performing defensive medicine. By its very nature, the unconscious practice of defensive medicine will not be reported by doctors.

In conclusion, defensive medicine is widely practiced by neurosurgeons in Turkey. Avoiding high-risk surgery, ordering unnecessary imaging studies, and referring patients to other hospitals are the most common forms of defensive medicine. Younger and less experienced neurosurgeons are more likely to practice defensive medicine in Turkey. These data should be taken into consideration by policy-makers and healthcare providers in order to design proper structural reforms for improving the quality of the healthcare system.
